# The relationship between proactive personality and college students’ short-form video addiction: A chain mediation model of resilience and self-control

**DOI:** 10.1371/journal.pone.0312597

**Published:** 2024-11-27

**Authors:** Shujie Wu, Zahid Shafait, Kaixin Bao

**Affiliations:** 1 Institute of Higher Education, Jinling Institute of Technology, Nanjing, China; 2 College of Education, Zhejiang Normal University, Jinhua, China; Shantou University Medical College, CHINA

## Abstract

**Background:**

With the explosive development of the short-form video (SFV) industry in recent years, the issue of SFV addiction among young people has attracted increasing attention from scholarship. Researchers have found that proactive personality is negatively associated with behavioral addiction. Additionally, SFV Apps tend to place users in a “filter bubble” to passively consume the pushed video content. Proactiveness could be an effective trait to resist the excessive use of SFVs. Thus, proactive personality may be a negative predictor of SFV addiction.

**Objective:**

The present study aims at investigating the relationship between proactive personality and SFV addiction among college students, and the psychological mechanism underlying the relationship, i.e., the mediating roles of resilience and self-control.

**Methods:**

Convenience sampling was adopted to conduct an online questionnaire survey among college students from 8 universities in 5 provinces of China. Proactive personality scale (PPS), brief resilience scale (BRS), Brief self-control scale (BSCS), and SFV addiction scale were applied in designing the questionnaire. A total of 560 valid questionnaires were obtained with ages ranging from 17 to 22 (19.32±1.14), among whom 40.18% were males and 59.28% females.

**Results:**

SFV addiction among college students was negatively correlated with proactive personality, resilience, and self-control. Proactive personality has significant direct effect on SFV addiction, and the other two variables play partial mediating roles including chain mediation between proactive personality and SFV addiction. The ratio of SFV addicts amounted to 23.57%, which was slightly higher compared to previous research findings.

**Conclusion:**

Proactive personality negatively predicts SFV addiction among college students and the mediators of resilience and self-control play partially mediating roles between proactive personality and SFV addiction.

## 1. Introduction

With the rapid development of mobile internet technology and the overwhelming popularity of smart devices, the short-form video (SFV) industry has experienced explosive growth in recent years. As of December 2023, the number of SFV users in China has reached 1.092 billion, among which SFV users account for 1.053 billion, with a usage rate of 96.4% [1]. The average daily usage time per user exceeds 2.5 hours, far exceeding that of other applications [2]. As pioneers and followers of new things, college students have quickly become loyal fans of various SFV products [3]. The group of college students generally has high user stickiness to SFV platforms, and even shows the phenomena of “addiction” [4].

SFV applications provide customized content for users based on an analysis of their preferences and employ big data and artificial intelligence to “anesthetize” the public by suppressing active thinking and time control ability [5]. When users passively express negative feedback by skipping over videos they do not like, it can significantly improve SFV sequential recommendation performance [6]. Although SFV platforms offer a wide variety of content, algorithmic recommendations may expose users to similar content and viewpoints repeatedly, leading to the so-called “filter bubble” effect and confining users within a narrow stream of information [7]. SFV applications place users in a situation where they tend to passively receive content pushed to them rather than actively seek out new topics or areas of interest. This passivity may lead to an increased dependence on the offered content, while diminishing the likelihood to actively explore diverse and valuable content. Users with high level of proactive personality tend to fight against the passivity, therefore it can be seen that SFV addiction is much related to lack of proactiveness or proactive personality in users.

A number of studies explored the external factors of SFV addiction, such as physical exercise [8], socio-technical and attachment perspective [9], parental neglect [10], and school burnout and social phobia [11]. Meanwhile, the internal factors mainly consist in individual motivation and psychological aspects, such as self-control and media literacy [12], and flow experience and cognitive lock-in [13]. Previous research found that that there was a certain correlation between behavioral addiction and personality traits [14]. However, the role of personality traits, particularly proactive personality, has remained largely unexplored in the context of SFV addiction. To address this research gap, this study aims at investigating the relationship between SFV addiction and proactive personality among college students, as well as the working psychological mechanism underlying the relationship. Unlike previous studies that primarily focus on external influences, this research shifts the focus to the intrinsic personality characteristics, i.e. proactive personality that may predispose individuals to SFV addiction. Therefore, this study uniquely contributes to the literature by investigating the impact of proactive personality on SFV addiction, an area that has not been adequately addressed in prior research.

Practically, this study can provide scientific references for educational authorities and universities to help prevent behavioral addictions and intervene in addictive behaviors among college students. Theoretically, this study can provide empirical evidence for research on the psychological factors influencing SFV addiction and revealed how proactive personality affects SFV addiction through a psychological regulatory mechanism.

## 2. Literature review

### 2.1 Definition of SFV and SFV addiction

SFVs refer to online videos that are published and shared through various new media platforms, with a duration ranging from a few seconds to several minutes [15]. SFVs are characterized by their fragmented nature, strong interactivity, diversified content, and deep integration into users’ daily lives [16]. Therefore, SFVs have become favored by the public in the internet era and number of users has experienced explosive growth in recent years.

Originally, addiction referred solely to substance addiction, which affects the mental conditions due to the uncontrollable excessive use of chemical substances, such as alcohol and drug addiction [17]. Griffiths proposed the issue of non-chemical substance addiction, namely the concept of behavioral addiction, such as pathological gambling, binge eating, internet addiction, shopping addiction, exercise addiction, sexual addiction, etc [18]. Scholars have further proposed the concept of technology addiction, which refers to users’ high psychological dependence to modern technology due to the excessive use of a certain technology, such as internet addiction and smartphone addiction [19]. Smartphone addiction is a type of non-biochemical behavioral addiction related to human-machine interaction, which falls in the category of technology addiction [18]. Internet addiction or smartphone addiction essentially have the following similar characteristics: (1) excessively long duration or high frequency of internet or smartphone use; (2) negative physical and psychological effects due to overuse of the internet or smartphone; (3) chronic and cyclical characteristics of these negative effects [20]. As one of smartphone addictions, SFV addiction was defined in this study as: a chronic or cyclical state of obsession caused by repeatedly using SFV apps, which results in intense and persistent feelings of need and dependence to SFVs.

### 2.2 Proactive personality and SFV addiction

As one of the positive psychological qualities, proactive personality has been receiving more and more attention to promote individual physical and mental development. It is an important personality trait that is distinct from the Big Five personality traits [21]. Proactive personality refers to a stable tendency of an individual to take initiative actions to influence their surrounding environment. Individuals with a proactive personality are not constrained by their environment and are skilled at identifying opportunities to change their situation [22]. Individuals with high proactive personality traits are adept at identifying opportunities and taking action. They possess characteristics of self-initiation, action orientation, and perseverance; in contrast, those with low proactive personality traits find it difficult to recognize and seize opportunities for change, and often exhibit passive and shortsighted characteristics [23].

Proactive personality has attracted widespread attention from scholarship, with college students as an important research subject. Among the college student population, proactive personality was found that it has strong correlations with many aspects, such as academic performance and engagement [24] and systems thinking skills [25]. Some studies attributed behavioral addiction to proactive personality. Individuals who are proactive is expected to use the Internet continuously for various activities such as information about new products, services, and innovations, information about competitors, and so on. These activities would allow them to increase their knowledge, thus enabling them to take appropriate actions which would bring beneficial results [26]. Individuals with high levels of proactive personality tend to have a low dependence on internet socializing, but they are willing to use this form of communication as an extension of their real-life social interactions. On the other hand, individuals with low levels of proactive personality often struggle with self-expression and frequently experience social frustration. However, they also have strong social needs, and the more concealed nature of online socializing provides an outlet for these needs. As a result, this group tends to develop a strong reliance on internet social interactions. Therefore, there is a negative correlation between proactive personality and online socializing. Individuals with gambling problems might react more hastily in stressful situations and are also less likely to prevent the occurrence of stressors. Moreover, low preventive coping might deprive the individual from protective effects of social support against gambling disorder [27]. Previous results from clinical studies found associations between proactive coping and several psychological disorders. For example, proactive coping was reported to be negatively associated with symptoms of post-traumatic stress disorder among female college students. Proactive coping may be a protective factor in the development of gambling disorder [28]. As one type of behavioral addiction or disorder, it is thus reasonable to assume that proactive personality has a positive impact on regulation and mitigation of SFV addiction.

According to the above analysis, this study proposed the following hypothesis:

H1 Proactive personality negatively predicts SFV addiction.

### 2.3 Proactive personality, resilience, and SFV addiction

Resilience refers to the phenomenon where individuals can successfully cope with and positively recover from stress or adversity [29]. It is also defined as the ability of an individual to overcome unfavorable situations and adapt positively to setbacks [30]. Stress can exert significant effects on one’s motives to use SFVs for escape or coping [31], therefore resilience plays an important role in overcoming stress and in turn preventing SVF addiction. Previous studies have demonstrated that proactive personality can positively predict an individual’s resilience. Zhu & Li (2021) found that proactive personality facilitates employee resilience via increasing work-related promotion focus and perceived insider identity [32]. Eşkisu (2021) investigated the role of proactive personality in the relationship between parentification and psychological resilience and proved that proactivity has a statistically significant positive effect on psychological resilience [33]. Resilience mediates the relationship between personality dimensions and psychological functioning during stressful situations like the COVID-19 pandemic [34]. Employees’ proactive personality can significantly positively predict their overall level of psychological capital and its four dimensions, which are optimism, hope, resilience and self-efficacy [35]. Nguyen et al. (2016) conducted a study with a sample group of 269 employees and concluded that proactivity predicts psychological resilience at a statistically significant level [36]. The above literature review shows that proactive personality is positively associated with resilience.

Many studies revealed that resilience is negatively correlated with behavioral addiction, such as internet addiction and smartphone addiction. It can negatively predict internet addiction among college students [37], and vocational students [38]. When facing difficulties and challenges, the stronger resilience an individual has, the more easily the individual can recover or even grow stronger after experiencing trauma, and the less likely they are to fall into internet addiction [39]. Regarding internet addiction, resilience is not only a negative predictor but also a mediator. For example, resilience has a significant partial mediating effect on the prediction of internet addiction by life events [40]. Among college students, resilience plays a mediating role between smartphone addiction and mental health, therefore cultivating resilience in college students can help mitigate the adverse effects of smartphone addiction on their mental health [41]. As a behavioral addiction similar to internet addiction and smartphone addiction, SFV addiction is supposed to be negatively correlated to resilience.

Individuals with proactive personalities typically possess stronger resilience, and resilience can negatively predict SFV addiction. The above literature review gave rise to the following hypothesis: H2 Resilience plays a mediating role between proactive personality and SFV addiction.

### 2.4 Proactive personality, self-control, and SFV addiction

Self-control is an important positive psychological force that can directly influence behavior. Cultivation of self-control can fortify the youth against adverse behaviors. Self-control refers to the ability to change one’s reactions to conform to ideals, values, morals, and social expectations, and to maintain the pursuit of long-term goals [42]. High proactive personality tendency is helpful to promote the development of self-control among adolescents [43].

Self-control is a common approach to explain the psychological mechanism underlying smartphone addiction and internet addiction. It was found that the college students who do not believe they can control their own actions and outcomes tend to give up their responsibility for the outcomes and acknowledge their internet identities. Self-control is an important factor that influences college students’ internet addiction [44]. Stress has a significant impact on smartphone addiction, and self-control can modulate the effect of stress on smartphone addiction. When self-control ability is reduced, stress is more likely to lead to smartphone addiction [45]. Geng (2018) held that internet addiction is positively correlated with procrastination, and significantly negatively correlated with core self-evaluation and self-control [46]. Therefore, self-control can effectively reduce excessive smartphone usage [47], and individuals with poor self-control ability are more likely to overuse video streaming services [48]. As to SFV applications, the content recommendation strategy is based on artificial intelligent algorithm technology [49]. SFV applications can continuously recommend personalized videos, and attract users to keep watching. College students have more free time at their disposal, and those with poor self-control are more likely to engage in excessive use of SFV applications [12]. It can be seen that self-control is negatively positively associated with behavioral addictions.

Based on the above analysis, it can be found that individuals with proactive personalities possess more abundant self-control resources to regulate SFV addiction. Therefore, this study proposed the following hypothesis: H3 Self-control plays a mediating role between proactive personality and SFV addiction.

### 2.5 Resilience and self-control

According to the theory of self-control resources, self-control resources can be affected by stressful situations [50]. In the process of coping with adversity and stress, an individual’s self-control resources may run out, which can lead to self-control failures [51]. Meanwhile, the capacity of resilience should be accompanied by protective factors such as social support, self-control, the ability to take the advantage of social relations, being able to act proactively, cognitive flexibility, and problem-solving skills [52]. Stressful experiences have a profound impact on the brain, for this reason, stress can increase vulnerability to addiction [53]. Individuals with low resilience are unable to recover well under stress; [54] therefore, they may stay in prolonged inescapable stress, which causes continuous depletion of self-control resources and a decreased level of self-control. In contrast, individuals with stronger resilience can cope with difficulties in a more positive mindset and maintain lower stress levels in adversity with rational beliefs and flexible methods [55]. They have more self-control resources to maintain or enhance their self-control abilities when facing adversity. In summary, resilience may be able to mitigate the depletion of self-control resources, enhance an individual’s self-control ability, and thereby reduce the likelihood of SFV addiction.

Considering the above literature review, this study proposed the following hypothesis:

H4 Resilience and self-control have chain mediating effects in the relationship between proactive personality and SFV addiction.

### 2.6 Hypothesis and research model

**Fig 1:**
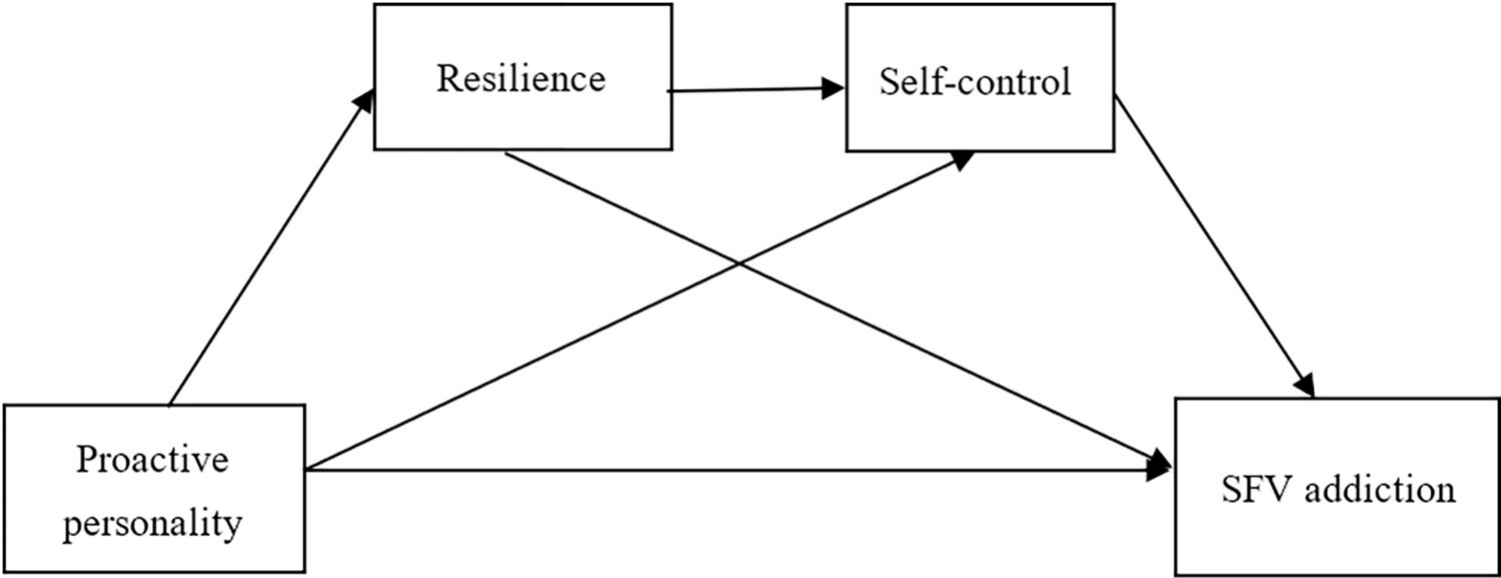
Research model.

This study aims at investigating the role of resilience and self-control in the relationship between proactive personality and SFV addiction. Fig 1 Research model illustrates the research model and four hypotheses are proposed as below:

H1: Proactive personality negatively predicts SFV addiction.
H2: Resilience plays a mediating role between proactive personality and SFV addiction.
H3: Self-control plays a mediating role between proactive personality and SFV addiction.
H4: Resilience and self-control play a chain mediating role between proactive personality and SFV addiction.

## 3. Materials and methods

To find out the answer to the question, “what is the role of resilience and self-control in the relationship between proactive personality and SFV addiction among college students”, this study adopted the survey method based on quantitative data. An online questionnaire was distributed to the target college students from different areas of China to guarantee the generality and efficiency of data collection. The questionnaire was designed on the basis of four mature measurement tools that have been widely used in previous studies. The collected data was then tested regarding common method bias through Harman’s single-factor analysis, and analyzed to set up the structural equation modeling and visualize the direct and indirect effects of the variables. After that, the findings were discussed and compared with previous studies.

### 3.1 Participants

This study employed convenience sampling to conduct an online questionnaire survey among college students from 8 universities in Jiangsu Province, Zhejiang Province, Shanghai, Chongqing, and Yunnan Province, China in January 2024. Though the survey ended in January 2024, data collection process spanned over five months i.e., from September 2023 to January 2024. Online questionnaire distribution significantly mitigates the negative effects of convenience sampling because online distribution allows for a wider coverage of population and areas. A total of 603 questionnaires were collected. After excluding those with patterned responses or those completed in an excessively short time (i.e. less than 5 minutes), we obtained 560 valid questionnaires. Table 1 shows the demographic features of the participants. By gender, there were 225 males (40.18%) and 335 females (59.82%). By years, there were 106 seniors (18.93%), 135 juniors (24.11%), 142 sophomores (25.36%), and 177 freshmen (31.61%). Ages ranged from 17 to 22, with an average age of 19.32±1.14. The survey was conducted in accordance with the Declaration of Helsinki, and approved by the Ethics Committee of the College of Education at Zhejiang Normal University (Protocol Number: ZSRT2024133).

**Table 1:** Social demographic features of participants.

Variables	Category	Number	Percentage
Gender	Male	225	40.18%
	Female	335	59.82%
Grade	Freshmen	177	31.61%
	Sophomores	142	25.36%
	Juniors	135	24.11%
	Seniors	106	18.93%
Age	17	5	0.89%
	18	168	30.00%
	19	143	25.54%
	20	133	23.75%
	21	108	19.11%
	22	4	0.71%

### 3.2 Measures

The variable scales involved in this study are all well-established scales, which have been widely used in the context of college students in China.

#### 3.2.1 Proactive Personality Scale

Bateman et al. (1993) developed Proactive Personality Scale (PPS) in English with 17 items [22]. This study used the Chinese version of PPS [56] revised by Shang & Gan (2009). The scale has a unidimensional structure with a total of 11 items. Each item is rated on a 7-point scale (1 indicates strongly disagree, 7 indicates strongly agree). Higher scores indicate a higher tendency or level of proactive personality. The internal consistency achieved with this scale in the present study was *α* = 0.897. The confirmatory factor analysis showed that the scale in this study has good structural validity, i.e., χ^2^/df = 2.412, CFI = 0.974, TLI = 0.968, GFI = 0.965, RMSEA = 0.050.

#### 3.2.2 Brief Resilience Scale

The Brief Resilience Scale (BRS) developed by Smith et al. (2008) is rather concise with a total of 6 items, half of which are phrased positively and half negatively [57]. The BRS has been applied in practice by numerous researchers, and cross-cultural revisions of the BRS have been completed in countries such as Turkey, Germany, Spain, and Brazil. In China, Chen et al. (2020) localized the scale for the population of Chinese college students and confirmed its good reliability and validity [58]. The internal consistency of this scale in the present study was *α* = 0.886. The confirmatory factor analysis showed that the scale in this study has good structural validity, i.e., χ^2^/df = 2.933, CFI = 0.990, TLI = 0.983, GFI = 0.985, RMSEA = 0.059.

#### 3.2.3 Brief Self-Control Scale

Morean (2014) revised the Self-Control Scale to generate a simplified version Brief Self-Control Scale (BSCS) that includes two dimensions (self-discipline and pulse control) with a total of 7 items [59]. The BSCS has been translated into multiple languages and has been confirmed to have cross-cultural consistency in its reliability and validity. In China, Luo et al. (2021) localized the scale and confirmed its good reliability and validity in the context of Chinese middle school students, vocational students, and college students [60]. The internal consistency of this scale in the present study was *α* = 0.876. The confirmatory factor analysis showed that the scale in this study has good structural validity, i.e., χ^2^/df = 2.507, CFI = 0.993, TLI = 0.989, GFI = 0.987, RMSEA = 0.043.

#### 3.2.4 SFV Addiction Scale

Based on Liang Yongchi’s Mobile Phone Addiction Index (MPAI) [61], Qin et al. (2019) developed the SFV Addiction Scale for college students, which includes four dimensions: anxiety and feeling lost, withdrawal and escape, inability to control craving, productivity loss, with a total of 14 items [20]. It has been confirmed to have good reliability and validity in the context of Chinese college students. In this study, the overall Cronbach’s alpha coefficient is 0.904, with the dimension of anxiety and feeling lost at 0.856, the dimension of withdrawal and escape at 0.859, the dimension of inability to control craving at 0.886, and the dimension of productivity loss at 0.789. The confirmatory factor analysis showed that the scale in this study has good structural validity, i.e., χ^2^/df = 1.930, CFI = 0.984, TLI = 0.980, GFI = 0.968, RMSEA = 0.041.

### 3.3 Procedure

The questionnaire in this study was designed and distributed through an online questionnaire system (WJX), and was filled out anonymously. The data were collected in a cross-sectional manner. Initially, the research team sent the questionnaire link to a group of students and encouraged them to promote the questionnaire to more potential participants through their social networks within schools via WeChat or QQ. To encourage students’ participation, red envelops (i.e. cash bonus function in the Chinese Instant Messaging Apps) were sent in the WeChat groups or QQ groups. It takes approximately 10 minutes to complete the questionnaire. The informed consent was designed as the first question of the questionnaire and all respondents were free to reject this survey after reading the informed consent. Upon completion of the questionnaire, the responding data were automatically saved on WJX and can be exported.

### 3.4 Data processing and analyzing methods

IBM SPSS Statistics software v27.0 software was used to conduct descriptive statistics and correlation analysis on the collected data. After the structural equation modeling approach was established based on the theoretical foundation and research hypotheses, Mplus version 8.3 was employed to conduct validated factor analysis and structural equation modeling to explore the relationship between proactive personality and SFV addiction, as well as to test the mediating roles of resilience and self-control.

## 4. Results

### 4.1 Common method bias test

Due to the use of questionnaire survey method in this study, there may exist common method bias. Harman’s single-factor analysis is a method used in factor analysis to check for common method bias in survey data. The bias can inflate relationships among variables and lead to erroneous conclusions. When the bias is less than the critical value 40%, it indicates an acceptable bias. Therefore, Harman’s single-factor analysis was conducted. An unrotated principal component factor analysis was performed on all variables. The results showed that there were 8 factors with eigenvalues greater than 1, and the variance contribution rate of the first factor was 30.799%, which is less than the critical value of 40% [62]. Therefore, the survey does not have severe common method bias in the data of this study.

### 4.2 Comparative analysis of addicts and non-addicts

According to the SFV addiction scale [20], the criterion for identifying SFV addicts is stated as the following: If respondents answer affirmatively to 5 out of 7 specific questions, they can be diagnosed as SFV addicts; if answering affirmatively to 4 out of 7 specific questions, they have a serious tendency toward SFV addiction. The original seven questions are stated in Chinese and translated into English as below:

1. *You become anxious if you haven’t watched short videos for a while*.
2. *When you’re out of mobile signal range for a while*, *you worry about missing out on some exciting short videos*.
3. *When you’re feeling down*, *you watch short videos to improve your mood*.
4. *You find yourself spending more time watching short videos than you originally planned*.
5. *You never feel like you’ve spent enough time watching short videos*.
6. *You’ve tried to spend less time watching short videos but couldn’t make it*.
7. *You find yourself getting absorbed in watching short videos when you should be doing other important tasks*, *which causes you some trouble*.

In the present study, the respondents with serious addictive tendency were regarded as SFV addicts. Based on the criterion, 132 out of 560 subjects are identified as SFV addicts, accounting for 23.57%. An independent-sample t-test was then conducted to compare the three variables between non-addicts and addicts. As shown in Table 2, non-addicts and addicts demonstrated very significant differences in proactive personality (t = 6.404, p<0.001), resilience (t = 4.959, p<0.001), and self-control (t = 7.344, p<0.001). Compared with addicts, non-addicts have stronger proactive personality (4.692>3.938), stronger resilience (3.317>2.847), and stronger self-control (3.169>2.471). Addicts have lower points in all the three variables, which indicates that the SFV addiction is negatively associated with the three variables.

**Table 2:** Comparative variable analysis between addicts and non-addicts.

Variables	Non-Addicts (N = 428)		Addicts (N = 132)		t	p
	M	SD	M	SD		
Proactive personality	4.692	1.170	3.938	1.219	6.404	<0.001
Resilience	3.317	0.949	2.847	0.962	4.959	<0.001
Self-control	3.169	0.976	2.471	0.880	7.344	<0.001

### 4.3 Correlations of variables

The correlation coefficients for each variable (see Table 3) show that the correlations of proactive personality, resilience, and self-control with SFV addiction are respectively -0.408**, -0.360**, and -0.453**. This indicates that the three variables have significant negative correlations with SFV addiction. With respect to the correlations among three variables, proactive personality has significant positive correlation with resilience and self-control, i.e., 0.317** and 0.472; resilience has significant positive correlation with self-control. This means that proactive personality, resilience, and self-control have significant correlations with each other.

**Table 3:** Correlation of variables.

Variables	M	SD	1	2	3	4
1 Proactive personality	4.514	1.223	1			
2 Resilience	3.206	0.972	.317**	1		
3 Self-control	3.004	0.997	.472**	.362**	1	
4 SFV addiction	2.276	0.647	-.408**	-.360**	-.453**	1

**Notes:** M = Means, SD = Standard Deviations

**p < 0.01

Table 4 shows the results of t test for independent sample of the four variables. It can be found that gender does not have significant correlation with all the four variables with p>0.05. Therefore, gender was not considered as a covariate in this study.

**Table 4:** T test for independent sample of variables by gender.

Variables	Gender				t	p
	Male (N = 225)		Female (N = 335)			
	M	SD	M	SD		
Proactive personality	4.5442	1.21280	4.4936	1.23191	0.480	0.632
Resilience	3.1800	.99319	3.2244	.95881	-0.529	0.597
Self-control	2.9863	.97656	3.0164	1.01340	-0.350	0.727
SFV addiction	2.2676	.69729	2.2827	.61230	-0.271	0.787

### 4.4 Structural equation modeling

**Fig 2:**
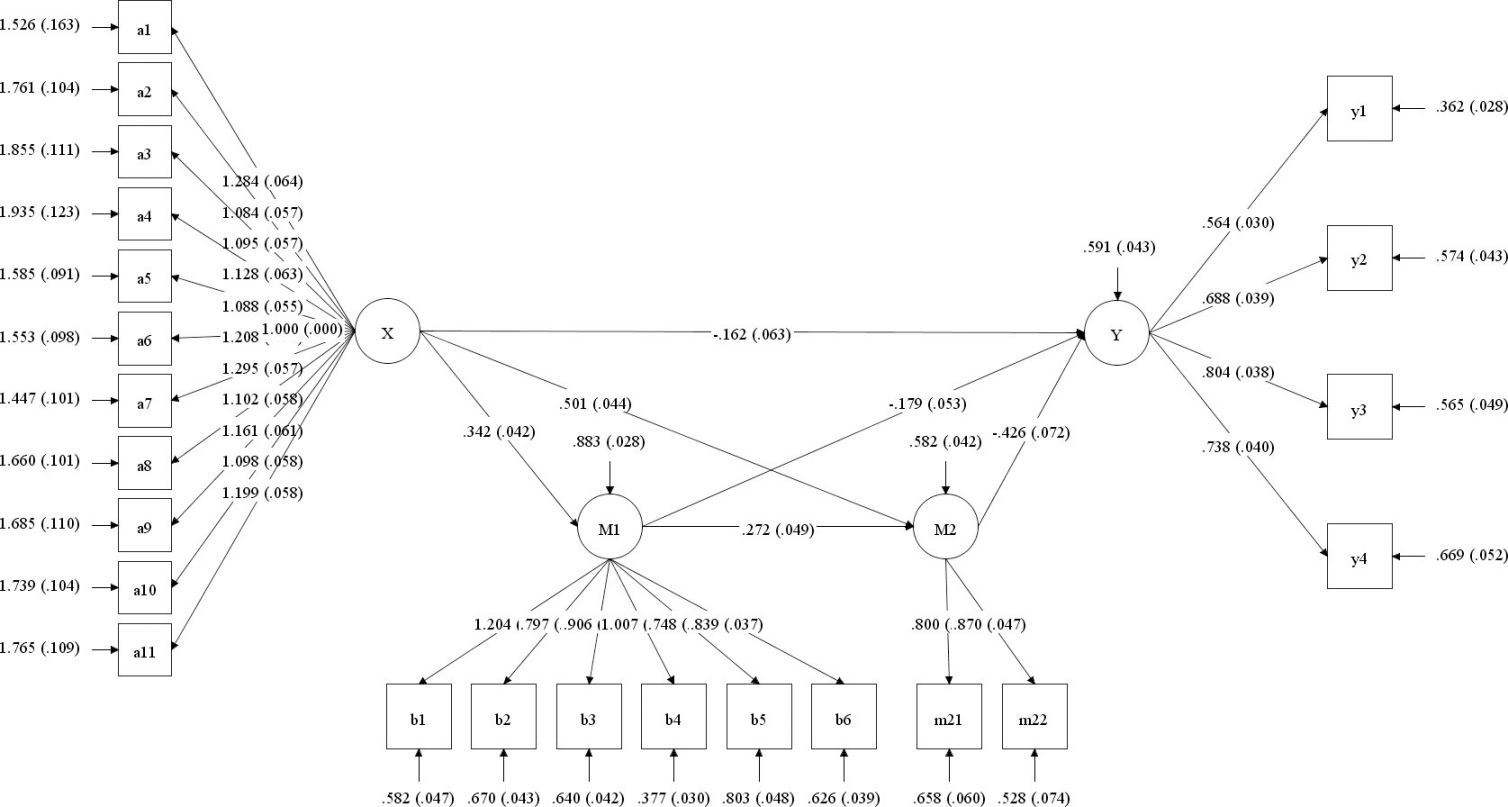
Structural equation model regarding the mediating effects of resilience and self-control between proactive personality and SFV addiction. **Notes**: All the coefficients are standardized estimates. **p < 0.01. **Abbreviations**: X = Proactive personality, Y = SFV addiction, M1 = Resilience, M2 = Self-control.

A structural equation model analysis was conducted with college students’ proactive personality as the independent variable, resilience and self-control as the mediating variables, and SFV addiction as the outcome variable, as shown in Fig 2 Structural equation model regarding the mediating effects of resilience and self-control between proactive personality and SFV addiction. This model demonstrated a good fit with the following data: *χ2/df* = 1.36, CFI = 0.985, TLI = 0.983, RMSEA (90% CI) = 0.025 (0.018~0.032), SRMR = 0.030.

Using the bias-corrected percentile Bootstrap test for mediation effects, the results in Table 5 showed that the 95% confidence intervals for all indirect paths did not include zero, indicating that the mediation paths were significant. In the model, the total indirect effect was significant (*β* = -0.320, p < 0.001). The indirect effect via self-control (*β* = -0.217, p < 0.001) was greater than that via resilience (*β* = -0.063, p < 0.01) and that via the chain of resilience and self-control (*β* = -0.040, p < 0.001).

**Table 5:** Standardized effects from proactive personality to SFV addiction.

Type	Path	β	SE	Effect Size	95% CI
Indirect	X→M1→Y	-0.061**	0.020	12.79%	[-0.107, -0.026]
	X→M2→Y	-0.213***	0.041	44.65%	[-0.131, -0.056]
	X→M1→M2→Y	-0.040***	0.011	8.39%	[-0.067, -0.022]
	Total indirect	-0.314***	0.043	65.83%	[-0.406, -0.213]
Direct	X→Y	-0.162**	0.063	33.96%	[-0.290, -0.040]
Total		-0.477	—	—	—

Note

**p < 0.01

***p < 0.001

**Abbreviations**: X = Proactive personality, Y = SFV addiction, M1 = Resilience, M2 = Self-control

To better understand how much variance was explained independently by each construct in this mediation model, we calculated the ratio of indirect to total effects for each construct as the effect sizes in Table 5. It can be found that the direct effect can only independently explain 34.29% of the total effect between proactive personality on SFV addiction. However, the indirect effect can independently explain 65.71%, which consists of 44.56% (mediated by self-control), 12.94% (mediated by resilience), and 8.21% (mediated by the chain of resilience and self-control).

## 5. Discussion

SFV addiction has become a prevalent behavioral addiction among college students today, necessitating further attention and understanding from educational authorities and universities. This study examined the relationship between proactive personality and SFV addiction through the mediating effects of students’ resilience and self-control. Our results showed that, as expected, proactive personality negatively predicts SFV addiction and resilience and self-control partially mediates the relationship between proactive personality and SFV addiction.

### 5.1 Importance of SFV addiction problem

This study showed that the ratio of SFV addicts among college students amounted to 23.57%, higher than the ratio of 20% reported in the "*Research Report on Internet Use and Cyber Security among Chinese Adolescents*" in 2018, and slightly higher than the result of 21.6% in Li et al.’s (2021) study on SFV addiction among Chinese college students [63]. This indicates that with the rapid growth of the SFV industry in recent years, the usage rate of SFVs among college students has also been rapidly increasing and the problem of SFV addiction has become worse. If this ratio is multiplied by the base number of college students throughout China, SFV addicts can constitute a huge population. SFV addiction has been an essential issue that poses considerable threats to students’ psychological well-being. Therefore, it is worth more attention from all relevant parties including education authorities, schools, families, and society.

In this study, the correlation coefficient between gender and SFV addiction was not significant, which is consistent with the finding of smartphone addiction research [64]. However, many studies argued that females experience higher levels of smartphone addiction compared to males. Such differences between smartphone addiction and SFV addiction by gender can be attributed to the following two reasons. First, the diversity of SFV content and platforms attracts a wide range of user groups, which may obscure the impact of gender on usage behavior. Second, individual psycho-social factors, such as social needs and self-control ability, may have a more direct impact on SFV addiction, rather than gender itself.

### 5.2 Proactive personality and SFV addiction

According to the research results, the direct effect of proactive personality on SFV addiction was significant (*β* = -0.167, p < 0.01) with an effect size of 34.29%. It proved H1 hypothesis, i.e., proactive personality negatively predicts SFV addiction. This finding was consistent with previous studies on the relationship between proactiveness and behavioral addictions. Proactive personality is negatively correlated with college students’ smartphone addiction, excessive screen time, and procrastination behaviors [65]. Individuals with proactive personality may be more effective at managing work-life balance and avoiding overwork, so workaholism was related negatively to proactivity [66]. Internet addiction was negatively predicted by proactiveness, i.e., individuals with a stronger proactive personality may exhibit less dependency on the Internet [67]. Therefore, it can be found that proactive personality has positive effects on preventing behavioral addictions including SFV addiction.

Proactive coping strategies refer to anticipatory actions and behaviors that individuals employ to prevent potential stressors or mitigate their impact before they occur. The strategies could include problem-focused coping (i.e., solving potential problems before they escalate), cognitive reappraisal (i.e., reframing how one interprets potential stressors in a positive way), building social support, anticipatory planning (i.e., anticipating challenges and making plans to address potential obstacles), and so on. College students report higher levels of psychological distress compared to the general population [68], and individuals with a proactive personality may use proactive coping strategies more effectively [69] to mitigate anxiety and distress. Proactive coping strategies significantly contribute to better psychological functioning by using resources effectively [70]. For example, it was found that proactive coping was negatively associated with gambling disorder among young gamblers [71]. Therefore, proactive individuals are more likely to engage in proactive coping, which prepares them to handle potential stressors before they become unmanageable and prevents behavioral addictions.

### 5.3 Mediating roles of resilience and self-control

According to the research results, the total indirect effect was significant (*β* = -0.320, p < 0.001) with a total size of 65.83%, including the mediator of self-control, the mediator of resilience, and the chain of the two mediators. The results confirmed the partial mediation of resilience and self-control between proactive personality and SFV addiction.

The mediating effect of self-control between proactive personality and SFV addiction was very significant (*β* = -0.217, p < 0.001) with an effect size of 44.65%. It proved H3 hypothesis, i.e., self-control plays a mediating role between proactive personality and SFV addiction. This finding means that self-control can negatively predicts SFV addiction, which is consistent with previous studies [72, 73]. According to the strength model of self-control which conceptualizes self-control as a finite resource that can be depleted through use [42], self-control can be strengthened through consistent exercise and practice. Engaging regularly in tasks that require self-control can increase an individual’s overall self-control capacity over time. Therefore, it is necessary for schools to create practicing opportunities for students by assigning academic or social practicing tasks that require self-control.

The mediating effect of resilience between proactive personality and SFV addiction was significant (*β* = -0.063, p < 0.01) with an effect size of 12.79%. It proved H2 hypothesis, i.e., resilience plays a mediating role between proactive personality and SFV addiction. This finding reveals that resilience can negatively predicts SFV addiction, which is consistent with previous studies regarding behavioral addictions, for example, resilience as a predictor of Internet addiction [38, 39, 74]. Therefore, resilience is an important protective factor against internet addiction in adolescents [74]. Individuals with poor resilience are weak in resisting adversity and stress, which makes them more susceptible to Internet addiction. Perceived stress significantly triggers short-form video addiction [75]. Individuals with stronger resilience tend to manage their emotions better and actively solve problems, thereby reducing the tendency to escape reality and indulge in SFVs.

The chain mediating effect of resilience and self-control is very significant (*β* = -0.040, p < 0.001) with an effect size of 8.39%. It proved H4 hypothesis, i.e., resilience and self-control play a chain mediating role between proactive personality and SFV addiction. This finding reveals that resilience are positively associated with self-control, which is consistent with previous studies. For example, individuals with stronger resilience scores have stronger self-control and are less likely to develop smartphone dependency [76], self-regulation, a component of self-control, is a significant index of adolescent resilience [77], and self-regulation is positively correlated with resilience and is a predictor of resilience in Spanish youth [78]. From the perspective of conservation of resources theory, mitigating the depletion of self-control resources in stressful situations can have a positive effect on an individual’s self-control ability [79]. Resilience is the ability to recover from stress [57]. Willpower strengthening exercises can effectively increase resilience and self-control, reducing psychological distress among university students [68]. Therefore, enhancing resilience training among college students can resist against behavioral addictions including SFV addiction. To mitigate the problem of SFV addiction among college students, joint efforts from schools, parents, and society to foster their resilience and self-control abilities.

Based on the above findings, the educational authorities, universities and families should focus on cultivating students’ proactive personality, resilience, and self-control ability in the education or intervention process of their mental health and psychological well-being. Further, literature in Chinese higher educational context has established that psychological and emotional management of personnel has profound effects on individual and organizational learning and performance [80–86]. Similarly, this study will motivate higher educational institutes to focus on mental well-being and resilience of stakeholders including students. This will help students establish effective self-management mechanisms and thereby reduce their excessive dependence on SFVs.

## 6. Limitations and future research

This study employed a cross-sectional research design, which explored the correlations between SFV addiction and college students’ proactive personality, resilience, and self-control. Whereas, this design cannot reflect the causal relationships or changing trends. Future research could adopt a longitudinal approach to further explore the dynamic relationships between SFV addiction and proactive personality and the dynamic mediating effects of resilience and self-control. The present study employed convenience sampling in a spatial scope of nine universities, which can introduce bias since it may not fully represent the broader population of college students. Future studies could employ random sampling or stratified sampling to improve the representativeness of the participants and expand the geographic scope (e.g., more universities from different provinces) to enhance the generalizability of the findings.

Additionally, the present study investigated the relationship between proactive personality and SFV addiction simply from the perspective of internal traits of individuals. Therefore, it is of much practical significance to conduct intervention study on SFV addiction, i.e., to explore how external social support (e.g. family care, peer influence, and institutional support) affects the relationship so that we can find more intervention measures to mitigate SFV addiction.

## 7. Conclusion

SFV addiction is a pathological factor that negatively influences college students’ academic gains and personal development. Resilience and self-control in the context of SFV addiction are vital personal resources, due to their involvement in the behaviors that favor a better adaptation and long-term individual trajectory. The results of this study find that proactive college students have stronger resilience and self-control resources to cope with stresses and resist SFV addiction. The two variables act as separate mediators and chain mediators in the relationship between proactive personality and SFV addiction. It can be concluded that proactive personality negatively predicts SFV addiction and the mediators of resilience and self-control play partially mediating roles in the relationship between proactive personality and SFV addiction. These findings also add empirical evidence to the existing literature in respect of the relationship between proactive personality and behavioral addictions.

This study has substantial theoretical and practical implications. Practically speaking, this study found that proactive personality, resilience, and self-control are key factors to reduce the risk of SFV addiction and promote mental health among college students. This provides a psychological science reference for educational authorities and universities in the prevention and intervention of students’ addictive behaviors. Theoretically speaking, this study provided empirical evidence for research on the psychological factors influencing SFV addiction and revealed how proactive personality affects SFV addiction through psychological regulatory mechanisms. In addition, it confirmed the mediating roles of resilience and self-control in behavioral addiction, enriching existing addiction theories and opening new directions for future research. Future research could adopt a longitudinal approach to further explore the dynamic relationship between SFV addiction and proactive personality and the dynamic mediating effects of resilience and self-control. It is of much significance to conduct intervention study on SFV addiction, i.e. to explore how external social support affects the relationship so that we can find more intervention measures to mitigate SFV addiction.

## Supporting information

S1 Data (XLSX)
